# Human amniotic fluid stem cells can improve cerebral vascular remodelling and neurological function after focal cerebral ischaemia in diabetic rats

**DOI:** 10.1111/jcmm.16956

**Published:** 2021-10-07

**Authors:** Ching‐Chung Liang, Steven W. Shaw, Yung‐Hsin Huang, Tsong‐Hai Lee

**Affiliations:** ^1^ Urogynecology Section Department of Obstetrics and Gynecology Chang Gung Memorial Hospital, Linkou Medical Center Taoyuan Taiwan; ^2^ College of Medicine Chang Gung University Taoyuan Taiwan; ^3^ Division of Obstetrics Department of Obstetrics and Gynecology Taipei Chang Gung Memorial Hospital Taipei Taiwan; ^4^ Prenatal Cell and Gene Therapy Group Institute for Women’s Health University College London London UK; ^5^ Stroke Center and Department of Neurology Chang Gung Memorial Hospital, Linkou Medical Center Taoyuan Taiwan

**Keywords:** amniotic fluid stem cell, diabetes, inflammation, rat, stroke, vascular remodelling

## Abstract

Diabetes causes vascular injury and carries a high risk of ischaemic stroke. Human amniotic fluid stem cells **(**hAFSCs) can enhance cerebral vascular remodelling and have the potential to improve neurological function after stroke in diabetic rats. Five groups of female rats were examined: (1) normal control, (2) type 1 diabetic (T1DM) rats induced by streptozotocin injection (DM), (3) non‐DM rats receiving 60‐minute middle cerebral artery occlusion (MCAO), (4) T1DM rats receiving 60‐minute MCAO (DM + MCAO) and (5) T1DM rats receiving 60‐minute MCAO and injection with 5 × 10^6^ hAFSCs at 3 h after MCAO (DM + MCAO + hAFSCs). Neurological function was examined before, and at 1, 7, 14, 21 and 28 days, and cerebral infarction volume and haemorrhage, cerebral vascular density, angiogenesis and inflammatory were examined at 7 and 28 days after MCAO. hAFSCs treatment caused a significant improvement of neurological dysfunction, infarction volume, blood‐brain barrier leakage, cerebral arterial density, vascular density and angiogenesis and a reduction of brain haemorrhage and inflammation compared with non‐treatment. Our results showed that the effect of hAFSCs treatment against focal cerebral ischaemia may act through the recovery of vascular remodelling and angiogenesis and the reduction of inflammation in ischaemic brain.

## 1 INTRODUCTION

Diabetes mellitus (DM) is health problem associated with multiple complications. DM patients fall into two pathogenetic categories^1^: type 1 diabetes (T1DM) due to insulin deficiency caused by autoimmune destruction of insulin‐producing pancreatic beta cells and type 2 DM due to defective insulin secretion from beta cells and peripheral insulin resistance.^1^ Exogenous insulin treatment can improve the life expectancy of DM, but almost all patients will eventually develop complications of micro‐ and macro‐vascular diseases.^2^ A meta‐analysis estimated the prevalence of DM was 28% in stroke patients, higher than general population.^3^ DM not only leads to high risk of ischaemic stroke but also causes poor restoration of neurological functions.^4^, ^5^ Mesenchymal stem cells (MSCs) can improve hyperglycaemia in animal and human trials based on their capabilities to restore endogenous pancreatic beta cell and immunomodulatory properties. However, despite MSCs offer hope for the treatment of T2DM, the effectiveness of MSCs for T1DM is inconclusive.^6^ Stem cells harvested from bone marrow and cord blood can mediate the therapeutic effects in animal stroke models, but the mechanism of action needs to be clarified.^7^, ^8^, ^9^ Previous studies found that treatment with bone marrow stromal cells (BMSCs) improved functional recovery after stroke in non‐diabetic rats^7^ but not in T1DM rats.^10^ However, bone marrow and cord blood stem cells are not easy to obtain, and it is difficult to generalize to clinical practice compared with amniotic fluid stem cells (AFSCs). Our recent research on bladder dysfunction in DM rats found that human AFSCs (hAFSCs) contributed to help recovery of pelvic vascular obstruction and bladder dysfunction.^11^, ^12^ The present study aimed to examine whether hAFSCs can improve neurological dysfunction, cerebral vascular remodelling, angiogenesis and inflammation after focal cerebral ischaemia in T1DM rats.

## 2 MATERIALS AND METHODS

### 2.1 Ethical approval

Animal experiments were approved by our Institutional Animal Care and Use Committee (No. 2018032203). Efforts were made to minimize suffering, reduced the number of animals used and utilized alternatives to in vivo techniques, if available. All procedures were performed in accordance with National Institute of Health Guide for the Care and Use of Laboratory Animals (NIH Publications No. 80–23) revised in 1996.

### 2.2 Animal preparation

Female ovariectomized Sprague‐Dawley rats (10–12 weeks old) were given tap water ad libitum and maintained at 21–23°C and 47% humidity with a 12‐hour light‐dark cycle and free access to standard laboratory chow and tap water. To induce ovariectomized status, rats underwent bilateral salpingo‐oophorectomy through a lower abdominal midline incision using sterile technique 2 weeks before middle cerebral artery occlusion (MCAO). One week after ovariectomizing, DM rat model was created by streptozotocin (STZ) injection. One week after STZ injection, DM rats underwent 60‐minute MCAO. Rats were randomly assigned to 5 groups including (1) normal controls, (2) STZ induced DM rats (DM), (3) non‐DM rats with 60‐minute MCAO (MCAO), (4) DM rats with MCAO (DM + MCAO), (5) DM + MCAO rats receiving 5 × 10^6^ hAFSCs treatment (DM + MCAO + hAFSCs).

**FIGURE 1:**
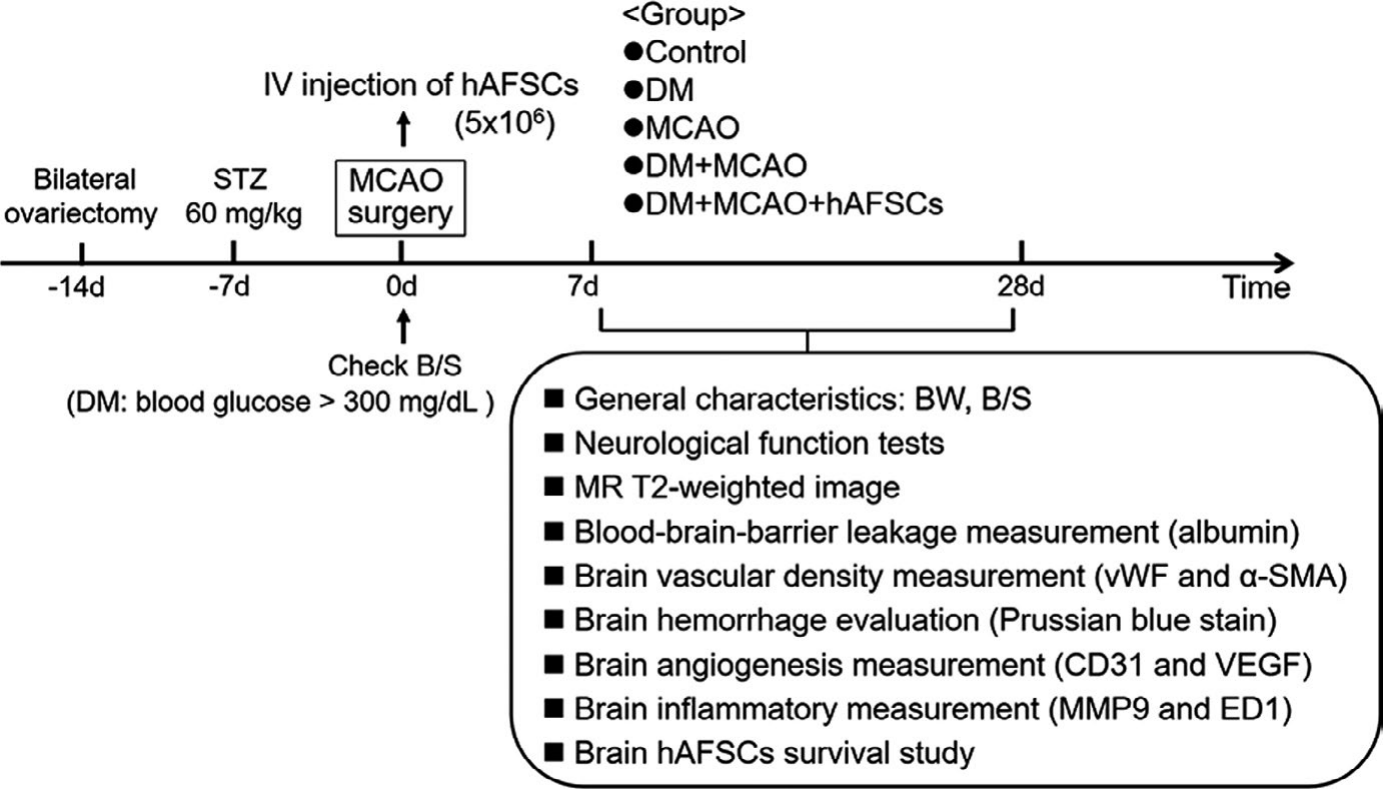
Schema of experimental procedure. STZ, streptozotocin; IV, intravenous; hAFSCs, human amniotic fluid stem cells; MCAO, middle cerebral artery occlusion; DM, diabetes mellitus; B/G, blood glucose; BW, body weight; CD31, cluster of differentiation 31; ED‐1, extra domain 1; MMP9, matrix metalloproteinase 9; MR, magnetic resonance; α‐SMA, alpha‐ smooth muscle actin; VEGF, vascular endothelial growth factor; vWF, Von Willebrand Factor

Body weight, blood glucose levels, neurological function, magnetic resonance (MR) imaging, and histological, immunohistochemical and immunofluorescent assessment were examined before MCAO and at 7 and 28 days after MCAO. Rats were euthanized at 7 and 28 days after MCAO and at similar timepoints if no MCAO. The experimental procedure is shown in Figure 1.

The reasons why the present study used female rats were first, our previous study^11^ examined the protective effect of hAFSCs against bladder dysfunction in a T1DM female rat model. For comparison, the present study used the same female rat with T1DM to investigate the effect of hAFSCs treatment on neurological dysfunction after focal cerebral ischaemia. Second, the authors are clinicians and experts in gynaecology and obstetrics, the use of ovariectomized female rats is in an attempt to understand and solve the similar neurological problems in postmenopausal female DM patients with cerebral stroke.

### 2.3 Induction of diabetes

DM was induced by single intraperitoneal injection of STZ, 60mg/kg diluted in 0.1 M citrate buffer solution, after 24‐hour fasting. Control group received single intraperitoneal injection of 0.1 M citrate buffer solution. Successful induction of DM was confirmed by measurement of blood glucose level at 7 days after STZ administration. Blood glucose levels were measured with ACCU‐CHEK advantage blood glucose monitoring system (Roche Diagnostics, Indianapolis, IN, USA). Rats with blood glucose level >= 300 mg/dl after fasting for 12 hours were deemed diabetic and suitable for study.

### 2.4 Induction of MCAO model

Transient right MCAO for 60 min was made according to our previous study.^13^ Under general anaesthesia with 3% isoflurane in a mixture of 30% O_2_ and 70% N_2_O, the proximal portion of external carotid artery was tightly ligated with a silk suture. A 20‐mm 4–0 nylon surgical thread was inserted from left external carotid artery into internal carotid artery to occlude middle cerebral artery. Left common carotid artery was then permanently ligated, and skin wound was temporarily closed. Anaesthesia was discontinued after these procedures were completed. After 60‐min occlusion of left middle cerebral artery, rat was anaesthetized again, and the wound was opened to remove nylon surgical thread to recanalize middle cerebral artery. In sham‐operated group, similar procedures were conducted except the ligation or occlusion of any cerebral vessel. Animals that did not exhibit left‐side weakness with upper‐limb dominance were considered failed MCAO and were excluded from analysis.

### 2.5 Magnetic resonance imaging

MR imaging was done according to our previous study.^13^ MR images were obtained using 7T Clinscan animal MR imaging system with a bore size of 30cm (Bruker, Ettlingen, Germany). A volume resonator with a diameter of 72mm was used for radio‐frequency transmission, and a 4‐channel phased array coil optimized for rat brain was used to receive signals. A T2‐weighted turbo spin‐echo sequence was applied to cover most of the brain regions of interest with 20 coronal slices using the following imaging parameters: TR/TE = 2920ms/38 ms, ETL = 7, slice thickness = 1 mm, matrix size = 256 × 256, in‐plane resolution = 0.148 × 0.148 mm^2^. From these images, 5 slices (2‐mm thickness) were selected based on a bregma of 0.4 mm and an interaural distance of 8.6mm for T2‐weighted imaging with set imaging parameters used for diffusion‐tensor imaging and dynamic susceptibility contrast MR imaging. The infarction area was measured separately in striatum and cortex of the 5 slices, and the infarction volume was calculated after multiplication by the distance.

### 2.6 Isolation and characterization of hAFSCs for treatment

hAFSCs were obtained using freshly collected amniotic fluid by routine amniocentesis from healthy pregnant donors in 15–20 gestational weeks. Institutional review board of our hospital approved this study (No. 201800452A3). Cells were cultured in StemPro® MSC serum‐free medium supplemented with 10% foetal bovine serum (Invitrogen, Carlsbad, CA, USA) and incubated at 37°C with 5% carbon dioxide. Culture medium was changed every 3–4 days. Specific surface antigens of hAFSCs were characterized by flow cytometry analyses as shown in our previous work.^14^ Cultured cells were trypsinized and stained with phycoerythrin (PE)‐conjugated antibodies against CD44, CD73, CD90, CD105, CD117 and CD45 (BD PharMingen, CA, USA). Thereafter, cells were analysed using Calibur flow cytometer (Becton Dickinson, Heidelberg, Germany). Passage 4–6 hAFSCs were collected and prepared to a final concentration of 5 × 10^6^ cells/0.3 ml in phosphate buffer solution (PBS). In hAFSCs‐treated groups, 5 × 10^6^ hAFSCs were administrated via tail vein at 3 h after MCAO. In DM + MCAO groups, 0.3 ml PBS were injected into tail vein at 3 h after MCAO. The timing of hAFSCs injection was based on our previous study.^14^ The treatment dose of hAFSCs was determined according to our previous studies that used stem cells to treat stroke in T1DM rats.^13^, ^14^

### 2.7 Neurological function tests

An investigator who was blinded to the experimental groups performed the neurological function tests including adhesive removal^7^ and foot‐fault test^15^ and evaluated the modified Neurological Severity Scores (mNSS)^7^ before MCAO, and at 1, 7, 14, 21 and 28 days after MCAO and at similar timepoints in no MCAO groups.

For adhesive‐removal somatosensory test, two small pieces of adhesive‐backed paper dots were used as bilateral tactile stimuli occupying the distal‐radial region on the wrist of each forelimb. The time to remove the stimulus from forelimbs was recorded based on five tests per day. Each test was separated from the previous test for at least 5 min.

For foot‐fault test, rats were placed on the grid for one minute, and the total number of placements of both forelimbs was counted. During this period, the number of foot‐fault errors in which the animals misplaced a forelimb causing falling through the grid was monitored, and the total number of errors for each forelimb was recorded.

The mNSS was a composite of motor, sensory, reflex, and balance tests and graded from 0 to 18. In the severity scores, one score point was awarded for the inability to perform the test or for the lack of a tested reflex; thus, the higher was the score, the more severe was the injury.

### 2.8 Histological, immunohistochemical and immunofluorescent assessment

Animals were euthanized at 7 and 28 days after brain MR imaging. Brains were dissected out, frozen in powdered dry ice and stored at −80°C. Coronal sections (20 μm) at the level of striatum covering infarcted brain region of 12 mm in length were prepared on a cryostat at −20°C and then transferred to glass microscope slides coated with saline (Muto Pure Chemical, Tokyo, Japan).

For immunohistochemical study, antibodies against α‐SMA (mouse monoclonal IgG, 1:200; Dako, Glostrup, Denmark) and vWF (rabbit polyclonal IgG, 1:400; Dako, Glostrup, Denmark) were used to identify arterial and vascular density. Antibody against albumin (FITC‐albumin, polyclonal, 1:500, Abcam Cambridge, MA, USA) was used to detect BBB leakage. Antibodies against matrix metalloproteinase 9 (MMP9, mouse monoclonal IgG, 1:50; Santa Cruz Biotechnology, CA, USA) and extra domain 1 (ED‐1, mouse monoclonal IgG, 1:100; Millipore, Temecula, CA, USA) were used to study the expression of pro‐inflammatory factor. Prussian blue stain was used to evaluate brain haemorrhage by measuring iron deposition (blue spots) outside of the vessels in brain parenchymal tissue.

Immunohistochemical study was done according to our previous method.^11^, ^12^, ^16^ Sections were first fixed for 10min in 4% paraformaldehyde and then rinsed with PBS. After blocking with Dako REAL peroxidase blocking solution (code S2023, DAKO Corp, Carpinteria, CA, USA) for 20 min, sections were washed and incubated with primary antibodies for 20 h at 4°C. Sections were then washed and incubated for 1 h with biotinylated secondary antibodies (1:200, Vector Laboratories, Burlingame, CA, USA). Staining was developed with 3, 3’‐ diaminobenzidine (DAB) plus hydrogen peroxide as the chromogen. Negative controls were performed without primary antibodies. Sections from each experimental group were put on the same slide to keep same incubation time for antibodies and chromogen.

For immunofluorescent study, antibodies against cluster of differentiation 31 (CD31, mouse monoclonal IgG, 1:50; Santa Cruz Biotechnology, CA, USA) and vascular endothelial growth factor (VEGF, rabbit polyclonal IgG, 1:100; Proteintech, Chicago, USA) were used to study microvessel density (CD31) and angiogenesis (VEGF). Antibody against hCD90 (1:200, BD Biosciences, San Jose, CA, USA) was used as the marker of mesenchymal stem cell.

Immunofluorescent study was done according to our previous method..^16^ Sections were first fixed for 10min in 4% paraformaldehyde and then rinsed with PBS. After blocking with 1% bovine serum albumin (BSA), sections were washed and incubated for 20 h at 4°C with primary antibodies, and then incubated for 1 hour with secondary antibody using Alexa‐fluor 488 or 594 (1:250, Invitrogen, Grand Island, NY, USA). Nuclear staining was performed with 4’,6‐diamidino‐2‐phenylindole (DAPI). Negative controls were performed without primary antibodies.

All quantification analysis was performed according to our previous study.^14^ Spot charge‐coupled device colour digital camera (Olympus DP72, Tokyo, Japan) was used to obtain immunohistochemistry images under a 20× objective (Olympus BX‐51, Tokyo, Japan) and immunofluorescence under Leica TCS SP8X confocal laser scanning microscope (Leica Microsystem, Heidelberg, Germany) with appropriate filters for FITC and DAPI. Camera was interfaced with Image‐Pro Plus Software (Media Cybernetics, Silver Spring, MD, USA).^17^ For statistical analysis, 5 sections from each brain with 2400 μm interval and 8 fields in ischaemic border zone (IBZ) of each section were examined. For albumin‐FITC and Prussian blue stain, the areas of positive staining were analysed.

### 2.9 Vascular and arterial density measurement

The IBZ adjacent to ischaemic core was identified using haematoxylin and eosin staining. To measure the vascular density, 8 fields of vWF immunostaining from IBZ in each brain section were digitized using a 20× objective via Image‐Pro Plus Software.^17^

The density of α‐SMA stained arteries in IBZ was measured^18^ in 5 brain sections with 8 regions in each section from the standard‐reference coronal section. The total number of positive α‐SMA staining vessels with wall diameter ≥10 μm in the 8 regions of IBZ was counted using Image‐Pro Plus Software (Media Cybernetics, Silver Spring, MD, USA).

### 2.10 Survival of hAFSCs in the brain

Immunofluorescent detection of hAFSCs in brain sections was performed by staining for human CD90 (hCD90) and DAPI. The survival of hAFSCs was assessed by counting the number of infiltrated hCD90 cells. The number of surviving cells in cortex and striatum was counted manually under 400× micrographs around infarction area in 5 sections and was summed as the total number of surviving hAFSCs per mm^3^.

### 2.11 Statistical analysis

Sample size calculation was done by using crude method based on law of diminishing return with the equation of E = total number of animals −total number of groups. After the calculation with (5 groups ×6 rats/group) − (5 groups) = 25, the number of 25 was more than 20, suggesting the sample size in this experiment could be more than necessary, and 6 rats were used for each group.^19^ Data were analysed with Prism 5 (GraphPad Software, La Jolla, CA, USA) and expressed as mean ± SD for continuous variables. Continuous data were compared among the groups by using one‐way analysis of variance. Tukey‐Kramer test was used for post hoc comparisons. To evaluate the effect of hAFSCs among groups, chi‐square test was performed with Fisher's exact test. Probability values of <0.05 were considered to be statistically significant.

## 3 RESULTS

### 3.1 hAFSCs treatment does not affect blood glucose level after stroke

DM and DM+MCAO rats had lower body weight and higher blood glucose levels than MCAO and control rats (Table S1). After hAFSCs treatment, body weight and blood glucose level did not significantly improve in DM + MCAO + hAFSCs rats at 7 and 28 days after MCAO compared with DM + MCAO rats.

### 3.2 hAFSCs treatment improves functional outcome after stroke

**FIGURE 2:**
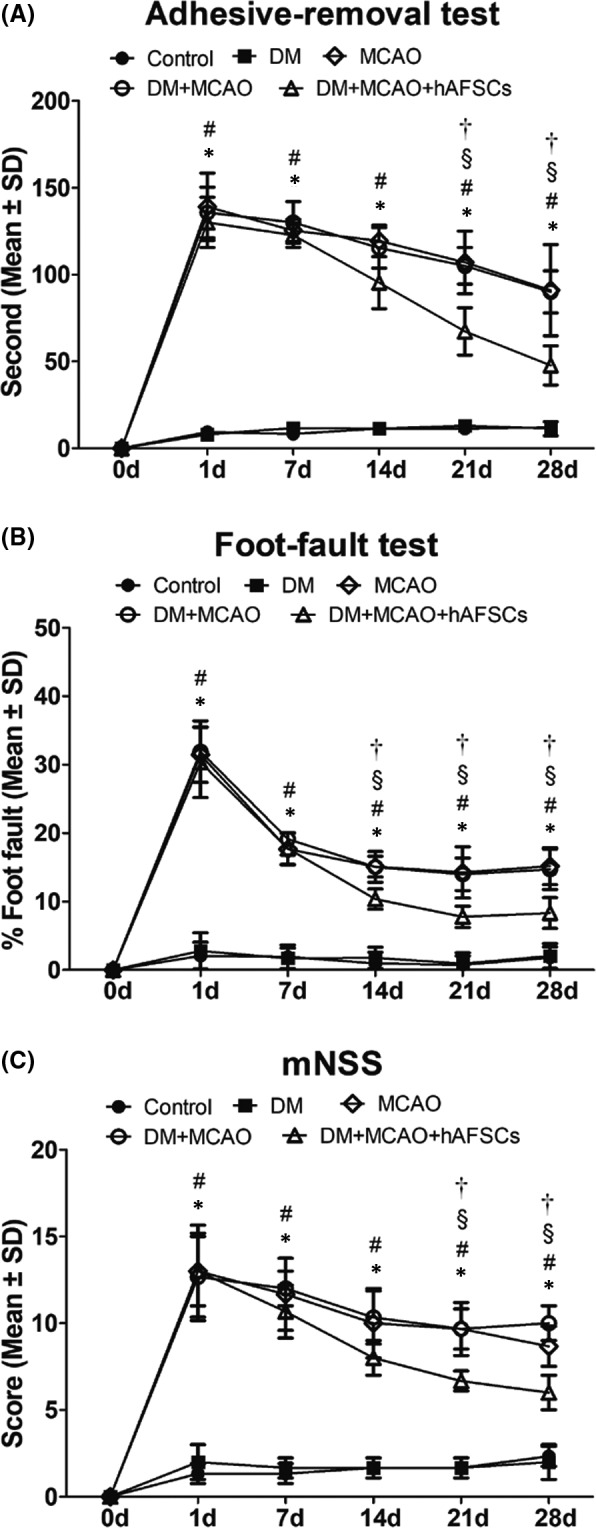
Neurological function tests including (A) adhesive‐removal test, (B) foot‐fault test and (C) modified neurological severity scores (mNSS) are evaluated before and 1, 7, 14, 21 and 28 days after MCAO. *: *p* < 0.001 vs. control, #: *p* < 0.001 vs. DM, §: *p* < 0.001 vs. MCAO, †: *p* < 0.001 vs. MCAO. *n* = 6 rats in each group. DM = diabetes mellitus, MCAO, middle cerebral artery occlusion; hAFSCs, human amniotic fluid stem cells

MCAO rats performed adhesive‐removal (Figure 2A), foot‐fault tests (Figure 2B) and mNSS (Figure 2C) poorly from 0 day to 28 days after stroke. Neurological function recovered better in DM + MCAO rats treated with hAFSCs than rats without treatment. Adhesive‐removal and mNSS scores improved at 21 and 28 days and foot‐fault tests improved at 14, 21 and 28 days after MCAO in rats treated with hAFSCs compared with those without treatment.

### 3.3 hAFSCs treatment decreases infarction volume after MCAO

**FIGURE 3:**
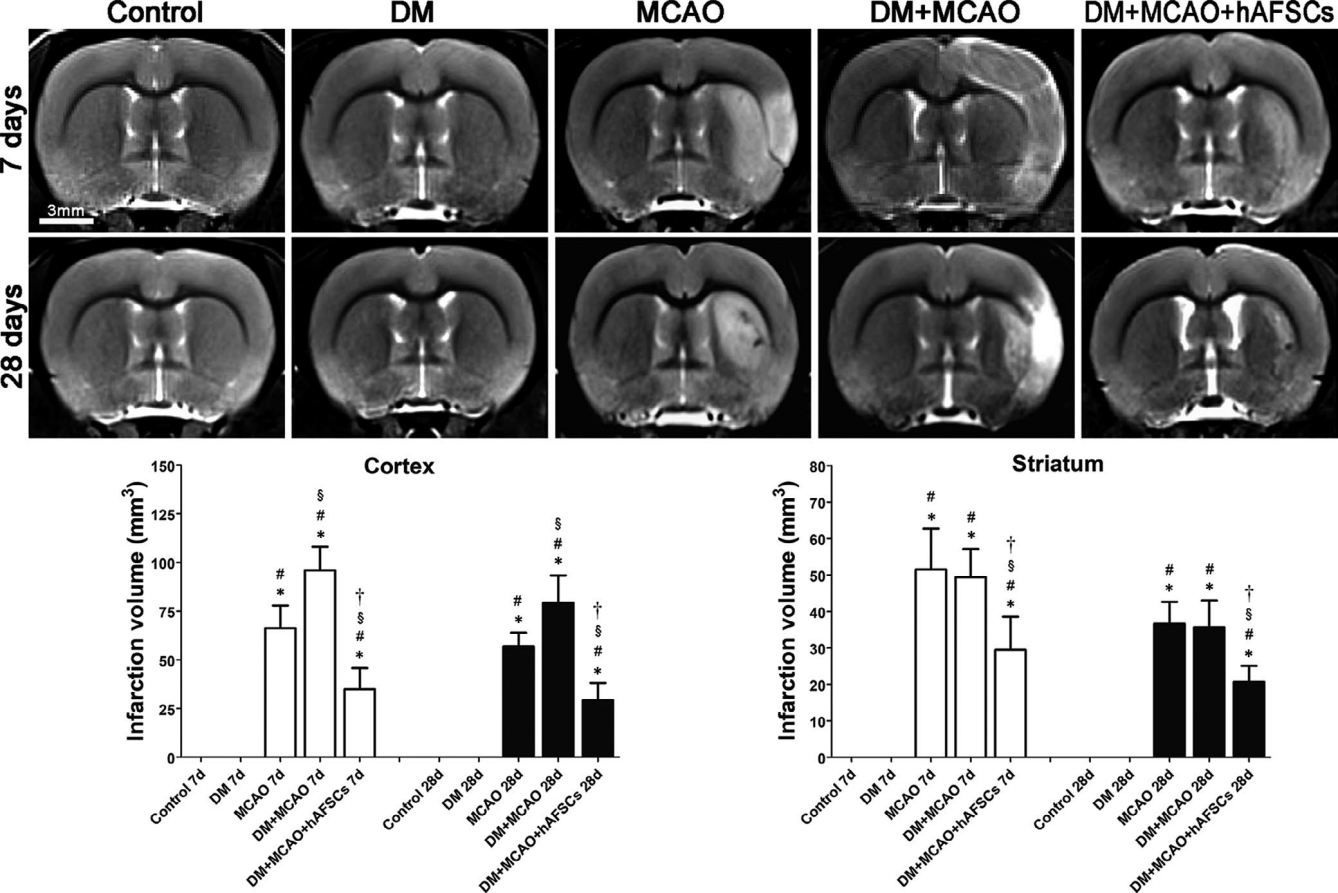
Brain magnetic resonance T2‐weighted images in control, DM, MCAO, DM+MCAO and DM+MCAO+hAFSCs groups. The infarction volume in cortex and striatum is significantly reduced in DM+MCAO+hAFSCs group compared with MCAO and DM+MCAO groups at 7 and 28 days after MCAO. In addition, the infarction volume in DM+MCAO rats is increased mainly in cortex compared with that in MCAO rats. §: *p* < 0.001 vs. MCAO and †: *p* < 0.001 vs. MCAO. *n* = 6 rats in each group. DM, diabetes mellitus; MCAO, middle cerebral artery occlusion; hAFSCs, human amniotic fluid stem cells

The infarction volume in cortex and striatum was significantly reduced in the DM + MCAO + hAFSCs group compared with MCAO and DM + MCAO groups at 7 and 28 days after MCAO (Figure 3). In addition, the infarction volume in DM + MCAO rats was increased mainly in cortex but not in striatum compared with that in MCAO rats.

### 3.4 hAFSCs treatment decreases BBB leakage, decreases brain haemorrhage, increases vascular density and improves vascular remodelling after MCAO

**FIGURE 4:**
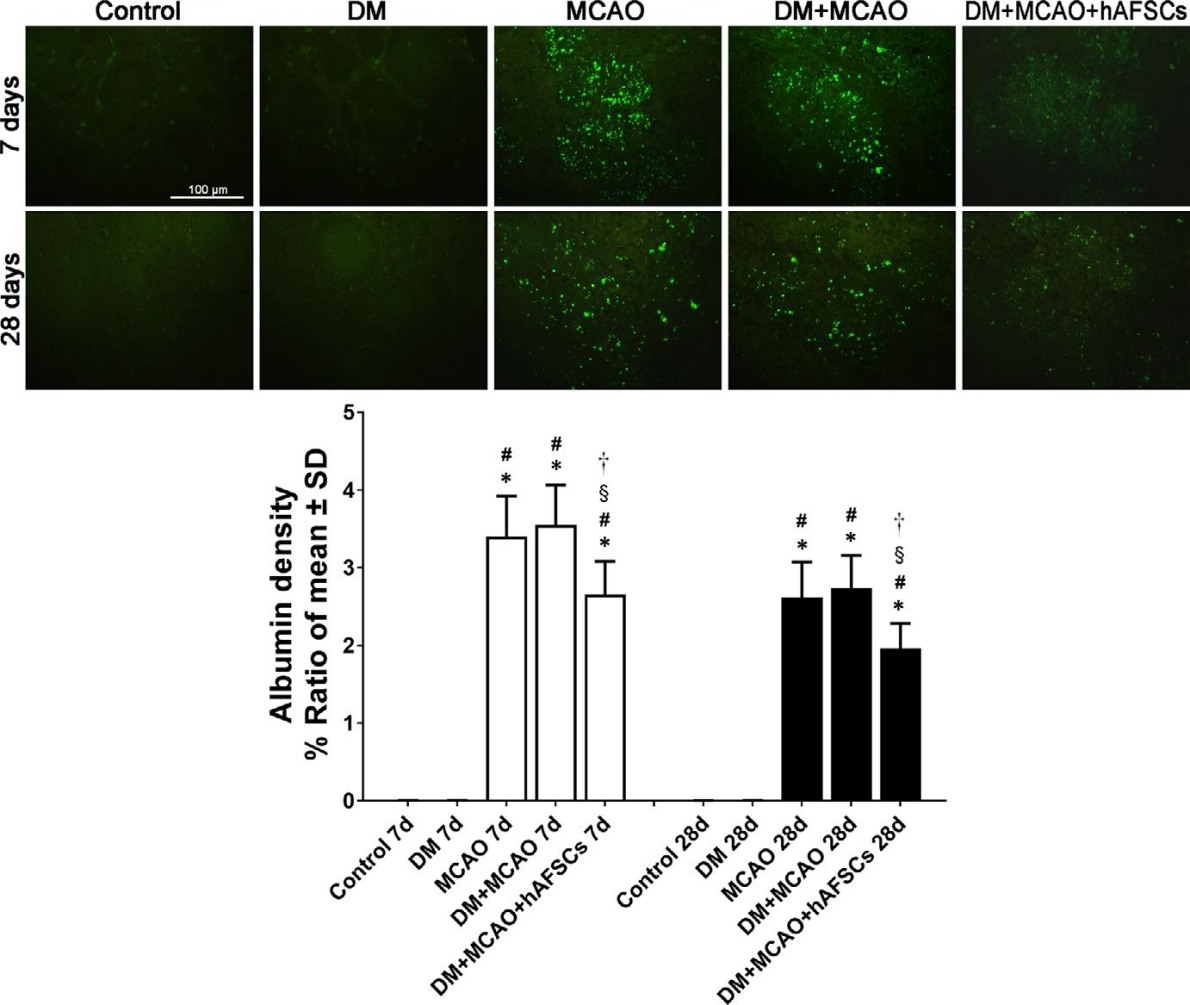
Immunofluorescent study of blood‐brain barrier (BBB) leakage. FITC‐albumin stain demonstrates that albumin density significantly decreases at 7 and 28 days after MCAO in DM+MCAO+hAFSCs group compared with MCAO and DM+MCAO groups. Bar =100 μm in sham panel. *: *p* < 0.001 vs. control, #: *p* < 0.001 vs. DM, §: *p* < 0.001 vs. MCAO and †: *p* < 0.001 vs. MCAO. *n* = 6 rats in each group. FITC‐albumin, Fluorescein isothiocyanate conjugated albumin; DM, diabetes mellitus; MCAO, middle cerebral artery occlusion; hAFSCs, human amniotic fluid stem cells

**FIGURE 5:**
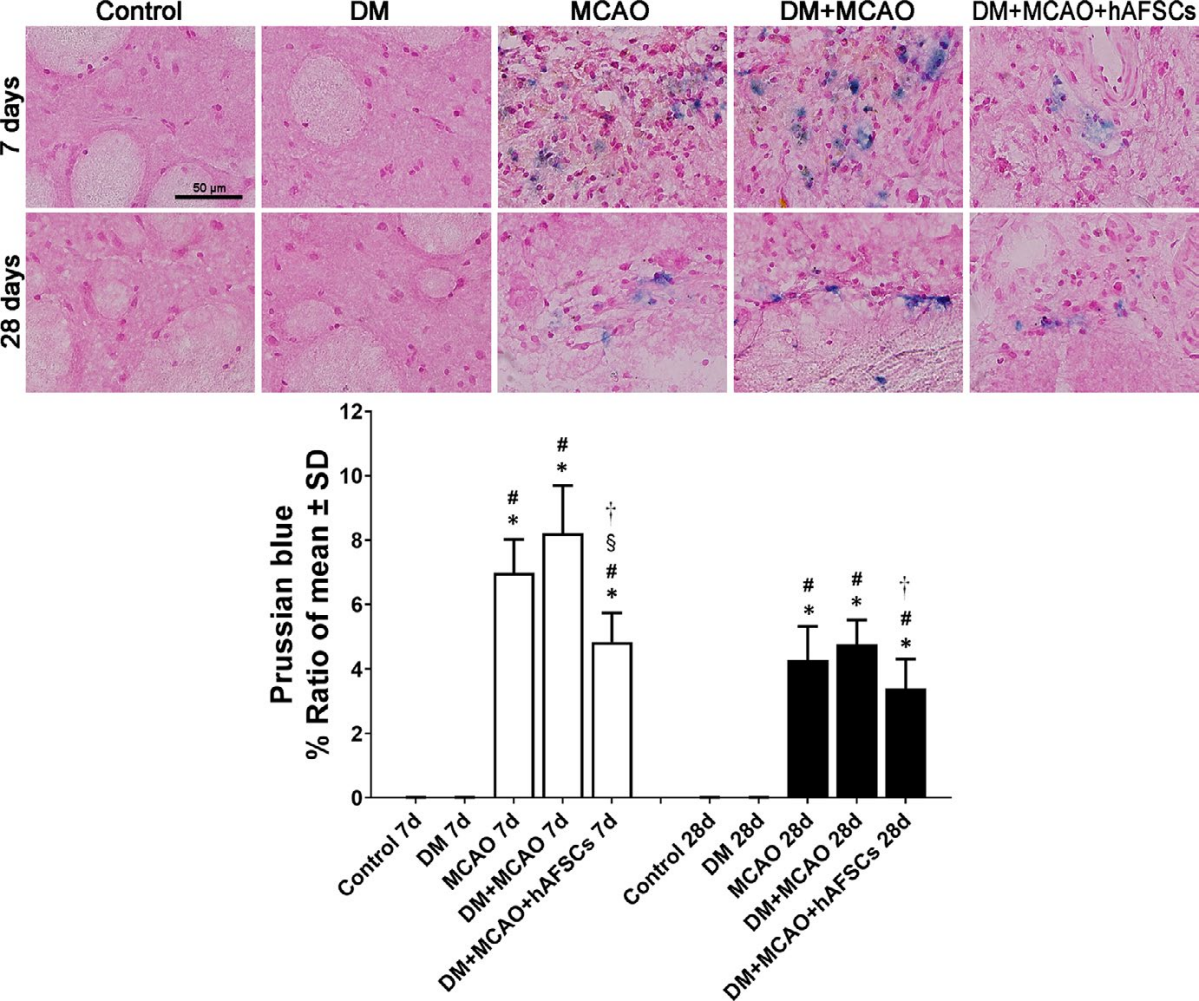
Prussian blue stain of brain haemorrhage in ischaemic brain tissue. Prussian blue stain demonstrates that the positive areas of Prussian blue are less in the DM+MCAO+hAFSC group compared with DM+MCAO group at 7 and 28 days after MCAO. Bar = 50 μm in sham panel. *: *p* < 0.001 vs. control, #: *p* < 0.001 vs. DM, §: *p* < 0.001 vs. MCAO and †: *p* < 0.001 vs. MCAO. *n* = 6 rats in each group. DM, diabetes mellitus; MCAO, middle cerebral artery occlusion; hAFSCs, human amniotic fluid stem cells

**FIGURE 6:**
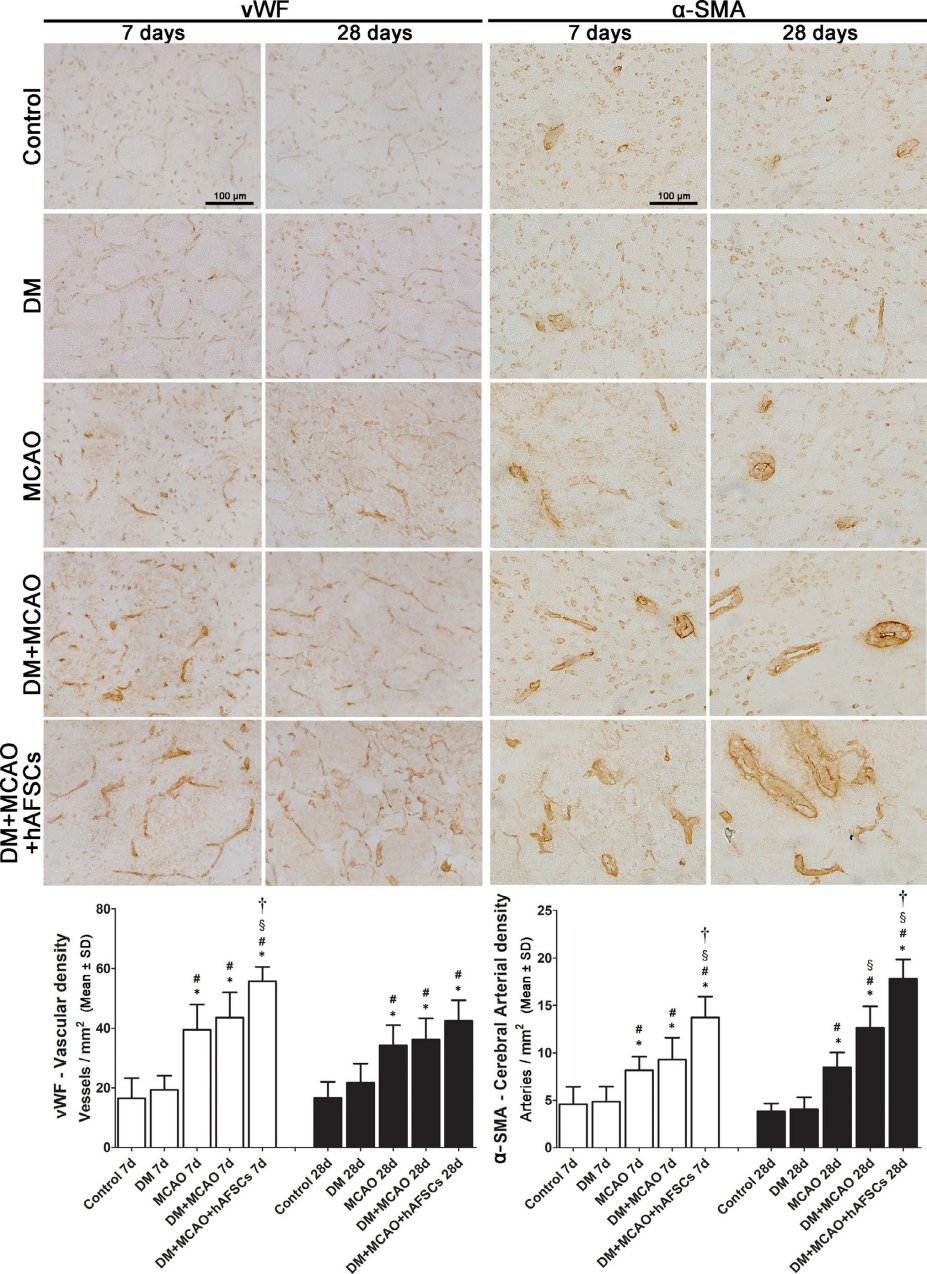
Immunohistochemical study of Von Willebrand factor (vWF) and alpha‐smooth muscle actin (α‐SMA) to measure the brain vascular density and arterial density. The DM+MCAO rats treated with hAFSCs have significantly increased vWF immunostaining (vascular density) at 7 days after MCAO and increased α‐SMA‐immunostaining (arterial density) at 7 and 28 days after MCAO compared with DM+MCAO rats with no hAFSCs treatment. Bar = 100 μm in sham panel. *: *p* < 0.001 vs. control, #: *p* < 0.001 vs. DM, §: *p* < 0.001 vs. MCAO and †: *p* < 0.001 vs. MCAO. *n* = 6 rats in each group. DM, diabetes mellitus; MCAO, middle cerebral artery occlusion; hAFSCs, human amniotic fluid stem cells

**FIGURE 7:**
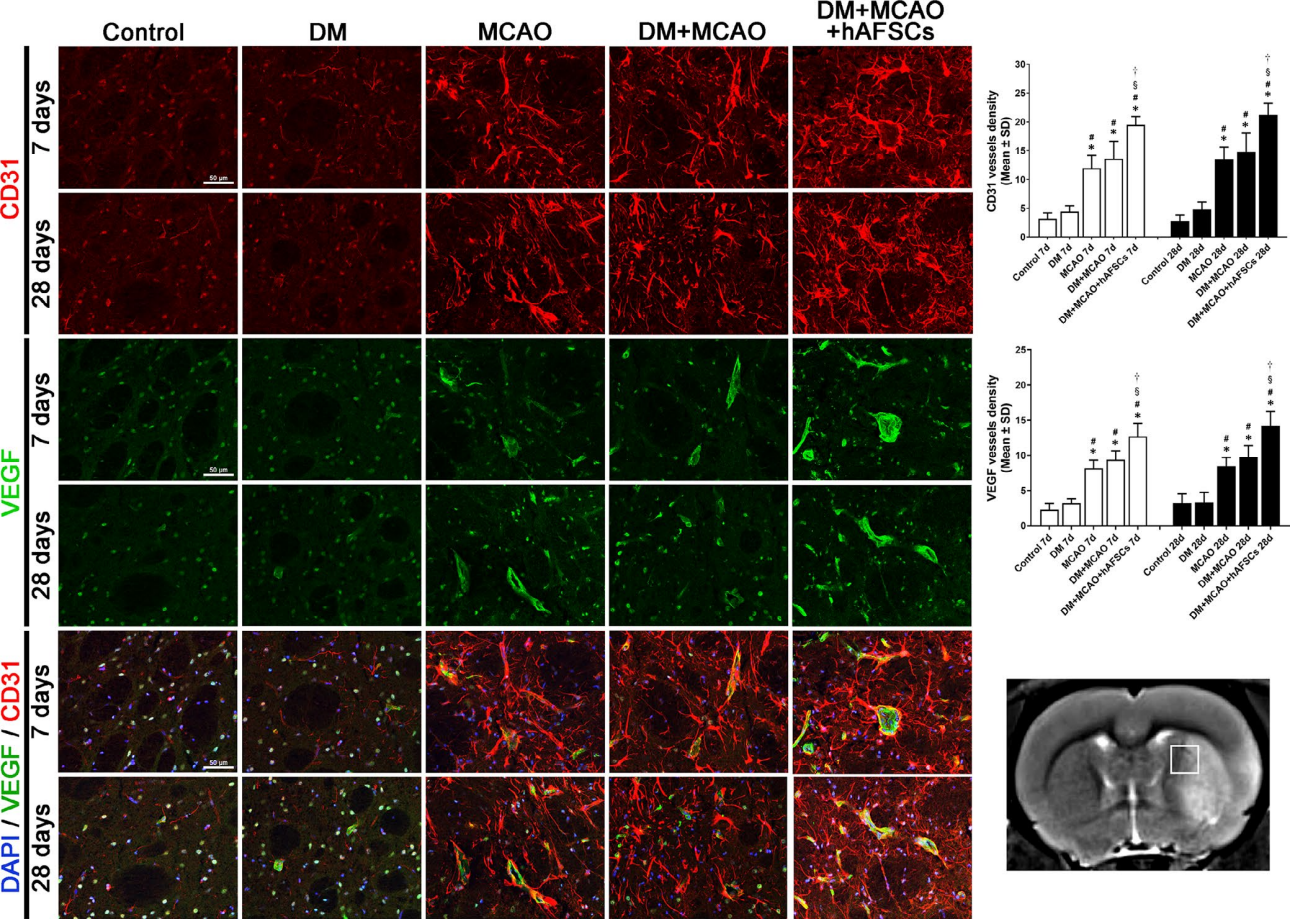
Immunofluorescent study of cluster of differentiation 31 (CD31) and vascular endothelial growth factor (VEGF) to measure brain microvessel density and vascular remodelling. Compared with MCAO and DM + MCAO groups, a higher level of microvessel density is detected in DM + MCAO+hAFSCs group at 7 and 28 days after MCAO. Also, a higher level of VEGF expression is detected in hAFSCs treatment group at 7 and 28 days after MCAO. Bar =100 μm in sham panel. *: *p* < 0.001 vs. control, #: *p* < 0.001 vs. DM, §: *p* < 0.001 vs. MCAO and †: *p* < 0.001 vs. MCAO. *n* = 6 rats in each group. DM, diabetes mellitus; MCAO, middle cerebral artery occlusion; hAFSCs, human amniotic fluid stem cells

The evaluation of FITC‐albumin level for BBB leakage (Figure 4), Prussian blue for brain haemorrhage (Figure 5), vWF and α‐SMA for vascular and arterial density (Figure 6) and CD31 and VEGF for vascular remodelling (Figure 7) was done in IBZ. FITC‐albumin stain demonstrated that albumin density significantly decreased at 7 and 28 days after MCAO in rats treated with hAFSCs compared with rats without hAFSCs treatment (Figure 4). The positive areas of Prussian blue stain at 7 and 28 days after MCAO were reduced after hAFSCs treatment compared with no hAFSCs treatment in DM + MCAO rats (Figure 5). In DM + MCAO rats, treatment with hAFSCs significantly increased vascular density (vWF) at 7 days and increased arterial density (α‐SMA) at 7 and 28 days after MCAO compared with no hAFSCs treatment (Figure 6). Compared with MCAO and DM + MCAO groups, there was a higher level of microvessel density (CD31) in DM + MCAO+hAFSCs group at 7 and 28 days after MCAO. Likewise, there was a higher level of VEGF immunofluorescence in hAFSCs treatment group at 7 and 28 days after MCAO (Figure 7). Overall, hAFSCs treatment significantly decreased BBB leakage and the risk of brain haemorrhage, but increased cerebral vascular and arterial density and improved vascular remodelling in IBZ compared with no hAFSCs treatment.

### 3.5 hAFSCs treatment decreases MMP9 and ED‐1 expression

The expression of MMP9 and ED‐1 significantly decreased at 7 and 28 days after MCAO when treated with hAFSCs compared with no hAFSCs treatment in DM + MCAO rats (Figure S1).

### 3.6 Survival of hAFSCs in the brain

The hCD90 staining showed some hCD90‐positive hAFSCs were seen at 7 days after hAFSCs treatment, and rare hCD90‐positive hAFSCs could be found at 28 days after hAFSCs treatment (Figure S2).

## 4 DISCUSSION

In the present study, we demonstrated for the first time that treatment with hAFSCs in T1DM rats could recover neurological dysfunction without reducing blood glucose levels and reduce infarction volume, decrease BBB leakage, decrease the risk of brain haemorrhage, and improve cerebral vascular remodelling, angiogenesis and inflammation after focal cerebral ischaemia.

Like embryonic stem cells, hAFSCs are multipotent and have the potential to differentiate into different cell types.^14^, ^20^ hAFSCs could be induced towards neural differentiation, and the specific markers of glial fibrillary acidic protein, beta‐III tubulin, nestin, CNPase, NeuN and synapsines could be detected.^16^, ^21^ hAFSCs have been reported to share similar characteristics to MSCs, including immune modulations, so hAFSCs may not exhibit immune rejection, unlike hematopoietic stem cells.^14^, ^16^, ^22^ It is likely that hAFSCs can differentiate and integrate into nervous tissue and have therapeutic potential in treating neurological disorders such as stroke and its complications. Our previous study on bladder dysfunction demonstrated that hAFSCs contribute to help recovery of bladder function in spinal cord injured rats and MCAO rats.^14^, ^16^ We found that hAFSCs have the capability to produce brain‐derived neurotrophic factor and possessed anti‐autoimmunity and anti‐inflammatory functions.^16^ The stem cells harvested from amniotic fluid have been shown to produce neurotrophic factors and release anti‐inflammatory cytokines to change the hostile environment related to secondary cell death in ischaemic brain.^23^ These findings may suggest hAFSCs can be a potential therapeutic agent for ischaemic injury.

Until now, few studies have focused on hAFSCs as a donor cell source for transplantation in ischaemic stroke. Treatment with hAFSCs may facilitate functional recovery in a rodent stroke model.^22^ In 60‐minute MCAO rats, hAFSCs labelled with iron nanoparticles were found to migrate to the ischaemic region after intravenous transplantation.^22^ Transplanted cells may provide support for cell surviving in penumbra area, offer protection from the toxic environment surrounding injured region and stimulate endogenous repair mechanisms such as neurogenesis, angiogenesis and immunomodulation.^22^, ^24^ Intravenous transplantation of rat AFSCs in adult rats at 30 days after MCAO showed that stem cell‐transplanted MCAO rats had significantly decreased infarction volumes at 60 days after MCAO compared to controls, which may help to facilitate the recovery of neurological functions.^23^

Preclinical results on cerebral infarction size and functional outcome after stroke are inconsistent in T1DM rats treated with stem cells.^10^, ^25^, ^26^ Treatment of BMSCs in T1DM rats at 24 h after MCAO failed to improve functional outcome and infarction volume.^10^, ^26^ However, human umbilical cord stem cells (hUCSCs) therapy performed 24 h after stroke in T1DM rats can improve functional outcome.^25^ Previously, we have reported that the infarction volume on T2‐weighted MR images obtained at 48 h after 60‐min MCAO was significantly smaller in hUCSCs treatment group than non‐treatment group.^13^ We inferred that hUCSCs may act through stabilization of cerebral haemodynamics and normalization of the expression of immediate early genes and brain‐derived neurotrophic factor after MCAO. The present study indicated that infarction volumes on MR image were reduced and functional outcomes were improved at 7 and 28 days after MCAO in hAFSCs treatment group compared with non‐treatment group in T1DM rats. Sibov et al^22^ reported that intravenous treatment of hAFSCs at 6 h after reperfusion can improve motor deficit and exploratory behaviour followed by reduction in infarction volume during the first 24 h after treatment with progressive improvement to 28 days.

DM can cause dysfunction of vascular endothelial cells,^27^ increase vascular permeability and impair recovery ability after ischaemic stroke,^28^ which were also reported in previous studies.^10^, ^25^, ^26^ The present study demonstrated that T1DM+MCAO rats had significant increase of BBB leakage and brain haemorrhage, but decrease of arterial and vascular density compared with controls. Permeable BBB can allow the infiltration of inflammatory factors from circulation into brain and exacerbate brain damage. In diabetic stroke animals, the increase of pro‐inflammatory factors can promote BBB disruption and lead to haemorrhagic transformation.^10^, ^29^ Failure of BMSCs treatment against stroke was reported being associated with increased BBB leakage, brain haemorrhage and inflammation in diabetic stroke rats.^10^, ^26^ The present study showed that hAFSCs treatment significantly increased vascular and arterial density but decreased BBB leakage and brain haemorrhage.

In addition, higher levels of CD31 (marker of microvessel density) and VEGF (growth factor in angiogenesis) expression in DM + MCAO rats can be found after hAFSCs treatment, indicating the possibility that improved angiogenesis may play an important role in the recovery after ischaemic stroke.^30^, ^31^ The increase in vascular remodelling may contribute to hAFSCs‐induced neurorestorative effects, but its underlying mechanism is not fully understood. Experimental study showed that hUCSCs treatment in T1DM stroke rats significantly promotes vascular and white matter remodelling with functional recovery after stroke, which may be attributed to elevating angiopoietin‐1 and suppressing inflammatory factors.^25^ Angiopoietins stimulate new blood vessel formation from preexisting vessels primarily in the IBZ,^32^ and can mediate vascular remodelling and promote axonal remodelling in ischaemic brain of T1DM rats.^33^

Previous studies demonstrated that treatment with BMSCs alone significantly increased the expression of angiogenin, MMP9 and ED‐1 in T1DM stroke rats, leading to the failure of BMSCs treatment against stroke.^10^, ^26^ However, the combination of BMSCs and Niaspan treatment can attenuate BMSCs‐induced inflammation in the ischaemic brain of T1DM rats.^26^ In the present study, hAFSCs treatment after cerebral stroke in T1DM rats significantly reduced the expression of pro‐inflammatory factor (MMP9) and ED‐1 (marker of macrophage) in ischaemic brain. These anti‐inflammatory effects of hAFSCs may partially contribute to improve the functional outcomes in T1DM rats.

Only some hCD90‐positive hAFSCs were seen in the brain sections of T1DM+MCAO rats at 7 days but not at 28 days after hAFSCs treatment. This result agrees with previous studies that revealed only 1% of injected stem cells could be detected in brain after intravenous administration of hUCSCs and human adipose tissue‐derived MSCs.^8^, ^34^ However, the present study found that although hAFSCs number decreased to nearly zero at 28 days (Figure S2), hAFSCs treatment could still improve neurological function at 28 days after stroke in T1DM rats. This phenomenon may suggest that the functional improvement after cerebral stroke may not be mainly related to the neurogenesis caused by hAFSCs differentiation but are of multimodal actions. hAFSCs has been reported to share similar characteristics to MSCs.^14^, ^16^, ^22^ The functional multipotency of MSCs demonstrates that besides the ability to shed secretomes and exosomes that may contain growth factors, anti‐inflammatory proteins, membrane receptors and microRNAs, MSCs may also undergo chemotactic migration towards developmental targets or inflammatory areas.^35^

The study using in vitro stroke model to investigate the signal transduction pathways activated by hAFSCs‐derived secretome^36^ revealed that microRNA analysis in the exosomal component of conditioned media could demonstrate overexpressed microRNAs that were involved in the regulation of coherent signalling pathways. MicroRNAs may mediate the neuroprotection through anti‐apoptotic and pro‐survival pathways.^36^ This pro‐survival effect was paralleled by the activation of neurotrophins, brain‐derived neurotrophic factor (BDNF)/ tropomyosin receptor kinase B (TrkB), and by the suppression of death pathways, p75/ c‐jun (JNK).^36^

The use of hAFSCs has some advantages. Bonaventura et al.^37^ compared the neural differentiation capability in four human stem cells from bone marrow, umbilical cord blood, human endometrium and amniotic fluid. They demonstrated that amniotic fluid is the most promising source of human multipotent cells because these stem cells are not affected by differentiation stimuli. Also, these stem cells have the ability to differentiate across all three germ layers by maintaining the non‐tumour forming properties, have the greatest differentiation potential towards neural cell lineage and proliferate more rapidly than post‐natal somatic cells and embryonic stem cells.

The present study has some limitations. First, we injected hAFSCs at one timing after MCAO. We examined the effect of hAFSCs given at 3 hours after MCAO based on the clinical guideline of intravenous recombinant tissue plasminogen activator treatment was advised to be used within 3–4.5 h after stroke onset.^38^ However, additional studies are warranted with hAFSCs administration at later time period after MCAO. Second, we examined neurological function and infarction volume at 7 and 28 days after MCAO. However, better results might be obtained if neurological function tests were performed at later period after treatment. Third, we did not perform immunosuppressive study. Our study and previous studies have revealed that injection of hAFSCs in animal models developed no immune rejection against transplanted tissue.^11^, ^12^, ^14^, ^16^, ^22^ hAFSCs share similar characteristics to MSCs, such as immune modulations, so hAFSCs may not exhibit immune rejection, unlike haematopoietic stem cells.^14^, ^16^, ^22^ Fourth, we did not use fibroblast or other cells as treatment control to compare with hAFSCs.

In conclusion, our data suggest that neurological dysfunction after MCAO can be improved by hAFSCs treatment in diabetic rats, which may be attributed to the improved vascular remodelling, angiogenesis and inflammation by hAFSCs treatment.

## CONFLICT OF INTEREST

The authors declare no conflict of interest.

## AUTHOR CONTRIBUTION

**Ching‐Chung Liang:** Conceptualization (lead); Data curation (equal); Formal analysis (equal); Funding acquisition (equal); Investigation (equal); Methodology (equal); Project administration (equal); Resources (equal); Software (equal); Supervision (equal); Validation (equal); Visualization (equal); Writing‐original draft (equal); Writing‐review & editing (equal). **Sheng‐Wen Steven Shaw:** Conceptualization (equal); Data curation (supporting); Formal analysis (supporting); Funding acquisition (supporting); Investigation (equal); Methodology (equal); Project administration (equal); Resources (equal); Software (equal); Supervision (equal); Validation (equal); Visualization (equal); Writing‐original draft (equal); Writing‐review & editing (equal). **Yung‐Hsin Huang:** Conceptualization (equal); Data curation (equal); Formal analysis (equal); Funding acquisition (equal); Investigation (equal); Methodology (equal); Project administration (equal); Resources (equal); Software (equal); Supervision (equal); Validation (equal); Visualization (equal); Writing‐original draft (equal); Writing‐review & editing (equal). **Tsong‐Hai Lee:** Conceptualization (lead); Data curation (equal); Formal analysis (equal); Funding acquisition (lead); Investigation (equal); Methodology (equal); Project administration (equal); Resources (lead); Software (equal); Supervision (equal); Validation (equal); Visualization (equal); Writing‐original draft (equal); Writing‐review & editing (equal).

## Supporting information

Fig S1

Fig S2

Table S1

## Data Availability

The data sets are available on reasonable request.

## References

[1] Xu G , Liu B , Sun Y , et al. Prevalence of diagnosed type 1 and type 2 diabetes among us adults in 2016 and 2017: Population based study. BMJ. 2018;362:k1497. 30181166 10.1136/bmj.k1497 PMC6122253

[2] Nathan DM . Long‐term complications of diabetes mellitus. N Engl J Med. 1993;328:1676‐1685. 8487827 10.1056/NEJM199306103282306

[3] Lau LH , Lew J , Borschmann K , Thijs V , Ekinci EI . Prevalence of diabetes and its effects on stroke outcomes: A meta‐analysis and literature review. J Diabetes Investig. 2019;10:780‐792. 10.1111/jdi.12932 PMC6497593 30220102

[4] Kamouchi M , Matsuki T , Hata J , et al. Prestroke glycemic control is associated with the functional outcome in acute ischemic stroke: The fukuoka stroke registry. Stroke. 2011;42:2788‐2794. 21817134 10.1161/STROKEAHA.111.617415

[5] Megherbi SE , Milan C , Minier D , et al. Association between diabetes and stroke subtype on survival and functional outcome 3 months after stroke: Data from the european biomed stroke project. Stroke. 2003;34:688‐694. 12624292 10.1161/01.STR.0000057975.15221.40

[6] Cho J , D'Antuono M , Glicksman M , Wang J , Jonklaas J . A review of clinical trials: Mesenchymal stem cell transplant therapy in type 1 and type 2 diabetes mellitus. Am J Stem Cells. 2018;7:82‐93. 30510843 PMC6261870

[7] Chen J , Li Y , Wang L , et al. Therapeutic benefit of intravenous administration of bone marrow stromal cells after cerebral ischemia in rats. Stroke. 2001;32:1005‐1011. 11283404 10.1161/01.str.32.4.1005

[8] Chen J , Sanberg PR , Li Y , et al. Intravenous administration of human umbilical cord blood reduces behavioral deficits after stroke in rats. Stroke. 2001;32:2682‐2688. 11692034 10.1161/hs1101.098367

[9] Vu Q , Xie K , Eckert M , Zhao W , Cramer SC . Meta‐analysis of preclinical studies of mesenchymal stromal cells for ischemic stroke. Neurology. 2014;82:1277‐1286. 24610327 10.1212/WNL.0000000000000278 PMC4001201

[10] Chen J , Ye X , Yan T , et al. Adverse effects of bone marrow stromal cell treatment of stroke in diabetic rats. Stroke. 2011;42:3551‐3558. 21940967 10.1161/STROKEAHA.111.627174 PMC3264886

[11] Liang CC , Shaw SS , Huang YH , Lin YH , Lee TH . Improvement in bladder dysfunction after bladder transplantation of amniotic fluid stem cells in diabetic rats. Sci Rep. 2018;8:2105. 29391467 10.1038/s41598-018-20512-z PMC5794746

[12] Liang CC , Shaw SS , Lin YH , Lee TH . Amniotic fluid stem cells ameliorate bladder dysfunction induced by chronic bladder ischemia in rat. Neurourol Urodyn. 2018;37:123‐131. 28605059 10.1002/nau.23316

[13] Liang CC , Liu HL , Chang SD , Chen SH , Lee TH . The protective effect of human umbilical cord blood cd34+ cells and estradiol against focal cerebral ischemia in female ovariectomized rat: Cerebral mr imaging and immunohistochemical study. PLoS One. 2016;11:e0147133. 26760774 10.1371/journal.pone.0147133 PMC4711929

[14] Liang CC , Shaw SW , Huang YH , Lin YH , Lee TH . Bladder transplantation of amniotic fluid stem cell may ameliorate bladder dysfunction after focal cerebral ischemia in rat. Stem Cells Transl Med. 2017;6:1227‐1236. 28186672 10.1002/sctm.16-0212 PMC5442832

[15] Rogers DC , Campbell CA , Stretton JL , Mackay KB . Correlation between motor impairment and infarct volume after permanent and transient middle cerebral artery occlusion in the rat. Stroke. 1997;28(10):2060‐2066. 9341719 10.1161/01.str.28.10.2060

[16] Liang CC , Shaw SS , Ko YS , Huang YH , Lee TH . Effect of amniotic fluid stem cell transplantation on the recovery of bladder dysfunction in spinal cord‐injured rats. Sci Rep. 2020;10:10030. 32572272 10.1038/s41598-020-67163-7 PMC7308393

[17] Chen J , Zhang ZG , Li Y , et al. Statins induce angiogenesis, neurogenesis, and synaptogenesis after stroke. Ann Neurol. 2003;53:743‐751. 12783420 10.1002/ana.10555

[18] Zacharek A , Chen J , Cui X , Yang Y , Chopp M . Simvastatin increases notch signaling activity and promotes arteriogenesis after stroke. Stroke. 2009;40:254‐260. 18927449 10.1161/STROKEAHA.108.524116 PMC2804086

[19] Charan J , Kantharia ND . How to calculate sample size in animal studies? J Pharmacol Pharmacother. 2013;4:303‐306. 24250214 10.4103/0976-500X.119726 PMC3826013

[20] De Coppi P , Bartsch G Jr , Siddiqui MM , et al. Isolation of amniotic stem cell lines with potential for therapy. Nat Biotechnol. 2007;25:100‐106. 17206138 10.1038/nbt1274

[21] Maraldi T , Bertoni L , Riccio M , et al. Human amniotic fluid stem cells: Neural differentiation in vitro and in vivo. Cell Tissue Res. 2014;357:1‐13. 24788911 10.1007/s00441-014-1840-x

[22] Sibov TT , Pavon LF , Cabral FR , et al. Intravenous grafts of human amniotic fluid‐derived stem cells reduce behavioral deficits in experimental ischemic stroke. Cell Transplant. 2019;28:1306‐1320. 31161782 10.1177/0963689719854342 PMC6767884

[23] Tajiri N , Acosta S , Glover LE , et al. Intravenous grafts of amniotic fluid‐derived stem cells induce endogenous cell proliferation and attenuate behavioral deficits in ischemic stroke rats. PLoS One. 2012;7:e43779. 22912905 10.1371/journal.pone.0043779 PMC3422299

[24] Rennie K , Haukenfrers J , Ribecco‐Lutkiewicz M , et al. Therapeutic potential of amniotic fluid‐derived cells for treating the injured nervous system. Biochem Cell Biol. 2013;91:271‐286. 24032676 10.1139/bcb-2013-0019

[25] Yan T , Venkat P , Ye X , et al. Hucbcs increase angiopoietin 1 and induce neurorestorative effects after stroke in t1dm rats. CNS Neurosci Ther. 2014;20:935‐944. 25042092 10.1111/cns.12307 PMC4180763

[26] Yan T , Ye X , Chopp M , et al. Niaspan attenuates the adverse effects of bone marrow stromal cell treatment of stroke in type one diabetic rats. PLoS One. 2013;8:e81199. 24303036 10.1371/journal.pone.0081199 PMC3841133

[27] Li W , Prakash R , Kelly‐Cobbs AI , et al. Adaptive cerebral neovascularization in a model of type 2 diabetes: Relevance to focal cerebral ischemia. Diabetes. 2010;59:228‐235. 19808897 10.2337/db09-0902 PMC2797926

[28] Capes SE , Hunt D , Malmberg K , Pathak P , Gerstein HC . Stress hyperglycemia and prognosis of stroke in nondiabetic and diabetic patients: A systematic overview. Stroke. 2001;32:2426‐2432. 11588337 10.1161/hs1001.096194

[29] Borlongan CV , Glover LE , Sanberg PR , Hess DC . Permeating the blood brain barrier and abrogating the inflammation in stroke: Implications for stroke therapy. Curr Pharm Des. 2012;18:3670‐3676. 22574981 10.2174/138161212802002841 PMC3411920

[30] Wang M , Li Y , Zhang R , et al. Adiponectin‐transfected endothelial progenitor cells have protective effects after 2‐hour middle‐cerebral artery occlusion in rats with type 2 diabetes mellitus. Front Neurol. 2021;12:630681. 33746885 10.3389/fneur.2021.630681 PMC7966523

[31] Asgari Taei A , Nasoohi S , Hassanzadeh G , Kadivar M , Dargahi L , Farahmandfar M . Enhancement of angiogenesis and neurogenesis by intracerebroventricular injection of secretome from human embryonic stem cell‐derived mesenchymal stem cells in ischemic stroke model. Biomed Pharmacother. 2021;140:111709. 34020250 10.1016/j.biopha.2021.111709

[32] Fagiani E , Christofori G . Angiopoietins in angiogenesis. Cancer Lett. 2013;328:18‐26. 22922303 10.1016/j.canlet.2012.08.018

[33] Yan T , Chopp M , Ye X , et al. Niaspan increases axonal remodeling after stroke in type 1 diabetes rats. Neurobiol Dis. 2012;46:157‐164. 22266016 10.1016/j.nbd.2012.01.001 PMC3335197

[34] Gomez‐de Frutos MC , Laso‐Garcia F , Diekhorst L , et al. Intravenous delivery of adipose tissue‐derived mesenchymal stem cells improves brain repair in hyperglycemic stroke rats. Stem Cell Res Ther. 2019;10:212. 31315686 10.1186/s13287-019-1322-x PMC6637493

[35] Teng YD . Functional multipotency of stem cells: Biological traits gleaned from neural progeny studies. Semin Cell Dev Biol. 2019;95:74‐83. 30822497 10.1016/j.semcdb.2019.02.002

[36] Castelli V , Antonucci I , d'Angelo M , et al. Neuroprotective effects of human amniotic fluid stem cells‐derived secretome in an ischemia/reperfusion model. Stem Cells Transl Med. 2021;10:251‐266. 33027557 10.1002/sctm.20-0268 PMC7848376

[37] Bonaventura G , Chamayou S , Liprino A , et al. Different tissue‐derived stem cells: A comparison of neural differentiation capability. PLoS One. 2015;10:e0140790. 26517263 10.1371/journal.pone.0140790 PMC4627815

[38] Lansberg MG , Bluhmki E , Thijs VN . Efficacy and safety of tissue plasminogen activator 3 to 4.5 hours after acute ischemic stroke: A metaanalysis. Stroke. 2009;40:2438‐2441. 19478213 10.1161/STROKEAHA.109.552547 PMC2725521

