# Supplementary figures and images for: Human amniotic fluid stem cells can improve cerebral vascular remodelling and neurological function after focal cerebral ischaemia in diabetic rats
Source: J Cell Mol Med. 2021 Oct 7;25(21):10185–96. doi: 10.1111/jcmm.16956 (PMC8572791; doi:10.1111/jcmm.16956)

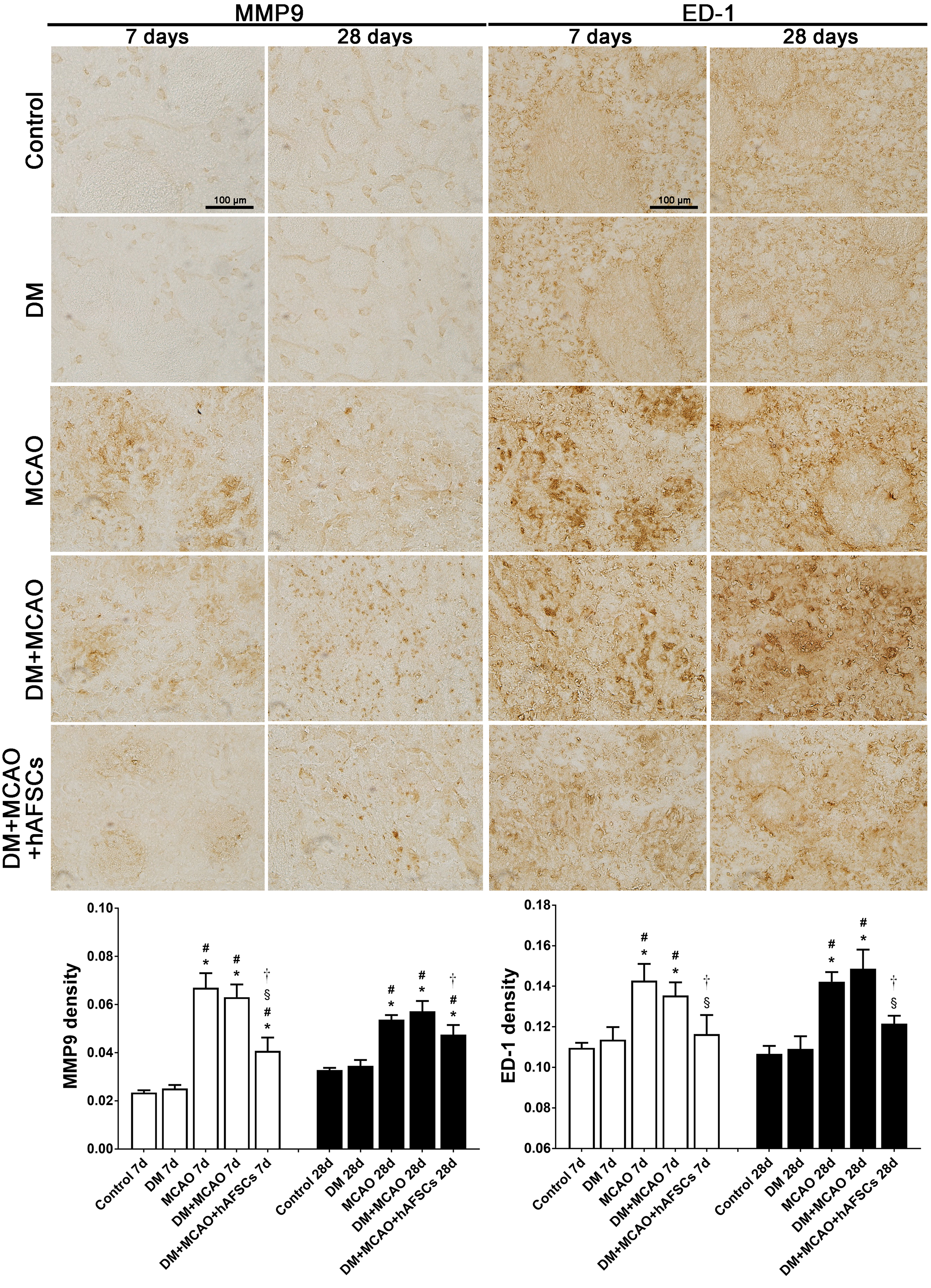

Supplement: Supplementary file 1 — Fig S1 [file JCMM-25-10185-s002.tif]

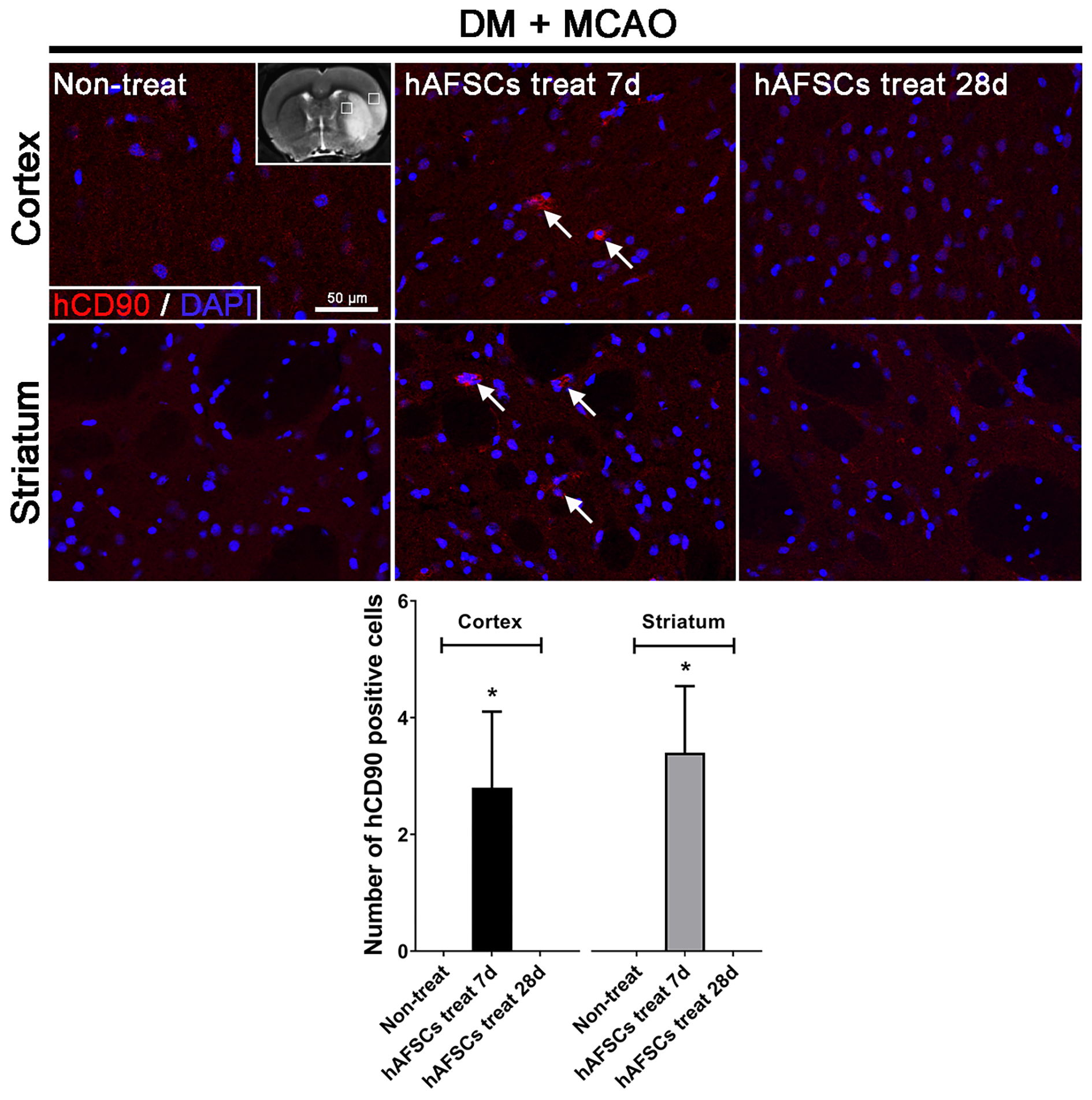

Supplement: Supplementary file 2 — Fig S2 [file JCMM-25-10185-s003.tif]
