# Supplementary material for: Human amniotic fluid stem cells can improve cerebral vascular remodelling and neurological function after focal cerebral ischaemia in diabetic rats
Source: J Cell Mol Med. 2021 Oct 7;25(21):10185–96. doi: 10.1111/jcmm.16956 (PMC8572791; doi:10.1111/jcmm.16956)
Supplement: Supplementary file 3 — Table S1 [file JCMM-25-10185-s001.docx]

**Supplementary Table 1. Changes in body weight and blood glucose levels before and after middle cerebral artery occlusion in the five groups**

|  | **Group (n = 6) (Mean ± SD)** | **Body weight (gm; Initial)** | **Body weight (gm; Final)** | **Blood glucose**  **(mg/dL; Initial)** | **Blood glucose**  **(mg/dL; Final)** |
| --- | --- | --- | --- | --- | --- |
| **7 days** | Control | 288.5 ± 10.7 | 303.1 ± 17.2 | 139.4 ± 10.5 | 159.4 ± 10.1 |
|  | DM | 284.2 ± 6.3 | 252.0 ± 20.6 * | 131.0 ± 9.9 | 408.3 ± 37.0 * |
|  | MCAO | 276.1 ± 12.6 | 274.0 ± 6.1 * | 144.0 ± 11.9 | 145.5 ± 9.5 # |
|  | DM+MCAO | 278.3 ± 13.7 | 248.0 ± 15.5 * | 141.4 ± 18.9 | 412.0 ± 32.7 *§ |
|  | DM+MCAO+hAFSCs | 283.4 ± 12.6 | 246.0 ± 10.0 * | 139.4 ± 9.3 | 443.8 ± 66.6 *§ |
|  | P value | 0.509 | < 0.001 | 0.839 | < 0.001 |
| **28 days** | Control | 293.5 ± 10.5 | 321.0 ± 13.8 | 134.8 ± 22.3 | 141.0 ± 5.8 |
|  | DM | 290.5 ± 24.7 | 265.4 ± 11.5 * | 143.0 ± 36.8 | 539.5 ± 21.9 * |
|  | MCAO | 291.4 ± 9.9 | 316.3 ± 10.7 # | 146.8 ± 14.3 | 143.3 ± 8.4 # |
|  | DM+MCAO | 288.6 ± 17.7 | 236.7 ± 32.5 *§ | 148.3 ± 22.2 | 517.3 ± 46.0 *§ |
|  | DM+MCAO+hAFSCs | 284.1 ± 12.7 | 257.8 ± 17.3 *§ | 136.0 ± 16.4 | 471.0 ± 51.9 *§ |
|  | P value | 0.868 | < 0.001 | 0.717 | < 0.001 |

* P < 0.001 vs. control (7 days and 28 days)

# P < 0.001 vs. DM (7 days and 28 days)

§ P < 0.001 vs. MCAO (7 days and 28 days)

n = 6 rats in each group

Initial body weight: body weight measured before MCAO in control, DM, MCAO, DM+MCAO and DM+MCAO+hAFSCs groups

Final body weight: body weight measured at 7 and 28 days after MCAO in control, DM, MCAO, DM+MCAO or DM+MCAO+hAFSCs groups

Initial blood glucose: blood glucose measured before MCAO in control, DM, MCAO, DM+MCAO or DM+MCAO+hAFSCs groups

Final blood glucose: blood glucose measured at 7 and 28 days after MCAO in control, DM, MCAO, DM+MCAO or DM+MCAO+hAFSCs groups

DM = diabetes mellitus, MCAO = middle cerebral artery occlusion, hAFSCs = human amniotic fluid stem cells.
